# Label-free nanoUPLC-MS^E^ based quantification of antimicrobial peptides from the leaf apoplast of *Nicotiana attenuata*

**DOI:** 10.1186/s12870-014-0398-9

**Published:** 2015-01-21

**Authors:** Arne Weinhold, Natalie Wielsch, Aleš Svatoš, Ian T Baldwin

**Affiliations:** Max Planck Institute for Chemical Ecology, Department of Molecular Ecology, Hans-Knöll-Straße 8, 07745 Jena, Germany; Max Planck Institute for Chemical Ecology, Mass Spectrometry/Proteomics Research Group, Hans-Knöll-Straße 8, 07745 Jena, Germany

**Keywords:** Intercellular fluid, Cysteine-rich peptides, Heterologous expression, Transgenic plants, Vacuum infiltration, Data-independent acquisition, Defensin, Lipid-transfer protein, Knottin

## Abstract

**Background:**

Overexpressing novel antimicrobial peptides (AMPs) in plants is a promising approach for crop disease resistance engineering. However, the *in planta* stability and subcellular localization of each AMP should be validated for the respective plant species, which can be challenging due to the small sizes and extreme p*I* ranges of AMPs which limits the utility of standard proteomic gel-based methods. Despite recent advances in quantitative shotgun proteomics, its potential for AMP analysis has not been utilized and high throughput methods are still lacking.

**Results:**

We created transgenic *Nicotiana attenuata* plants that independently express 10 different AMPs under a constitutive 35S promoter and compared the extracellular accumulation of each AMP using a universal and versatile protein quantification method. We coupled a rapid apoplastic peptide extraction with label-free protein quantification by nanoUPLC-MS^E^ analysis using Hi3 method and identified/quantified 7 of 10 expressed AMPs in the transgenic plants ranging from 37 to 91 amino acids in length. The quantitative comparison among the transgenic plant lines showed that three particular peptides, belonging to the defensin, knottin and lipid-transfer protein families, attained the highest concentrations of 91 to 254 pmol per g leaf fresh mass, which identified them as best suited for ectopic expression in *N. attenuata*. The chosen mass spectrometric approach proved to be highly sensitive in the detection of different AMP types and exhibited the high level of analytical reproducibility required for label-free quantitative measurements along with a simple protocol required for the sample preparation.

**Conclusions:**

Heterologous expression of AMPs in plants can result in highly variable and non-predictable peptide amounts and we present a universal quantitative method to confirm peptide stability and extracellular deposition. The method allows for the rapid quantification of apoplastic peptides without cumbersome and time-consuming purification or chromatographic steps and can be easily adapted to other plant species.

**Electronic supplementary material:**

The online version of this article (doi:10.1186/s12870-014-0398-9) contains supplementary material, which is available to authorized users.

## Background

Antimicrobial peptides (AMPs) are a diverse group of small, cationic peptides that can inhibit the growth of a broad range of microbes. They can be found in plants as well as in animals and have been shown to play an important role in defense and innate immunity [1,2]. The stable ectopic expression of AMPs in plants allows for the use of plants as biofactories or in the protection of crops against a wide range of pathogens [3,4]. A universal method that could verify *in planta* AMP stability and accumulation would allow for the rapid screening of different candidates to find novel AMPs for plant protection.

One of the first animal-peptides heterologously expressed in plants was cecropin B, a small AMP from the giant silk moth *Hyalophora cecropia*. Attempts to detect the peptide in transgenic tobacco and potato plants failed, indicating *in planta* instability [5,6]. Cecropin B has been shown to be extremely susceptible to endogenous plant peptidases and even modified versions of the peptide had half-lives of only few minutes when exposed to various plant extracts [7,8]. Finally, peptidases identified within the intercellular fluid of *Nicotiana tabacum* plants [9], were found to be responsible for peptide degradation, and remain a festering problem for the heterologous protein production in plants [10]. Recent studies repeatedly report peptide instabilities [3], which has become the main focus for the *de-novo* design of AMPs for plant protection [11,12].

Most AMPs share a number of features: they are very small (<10 kDa), highly cationic charged and have an even number of conserved cysteine residues (4, 6 or 8), which are connected by intra-molecular disulfide bridges [13]. Cysteine-free AMPs are rarely described in plants, and among these, mainly glycine-rich peptides showed a similar antimicrobial activity [14,15]. AMPs are typically produced as pre-proteins containing N-terminal signal peptides, essential for successful heterologous expression, as they avoid an undesired intracellular accumulation and allow the formation of disulfide bridges when passing through the endoplasmatic reticulum. The secretion and extracellular accumulation of AMPs is also a natural prerequisite for a plant to “poison the apoplast” and protect the intercellular space against the invasion by microbial pathogens [16].

The plant cell wall proteome (or secretome) is insufficiently studied, as the extraction of cell wall proteins can be challenging [17,18]. Secreted proteins can bind the polysaccharide matrix or other cell wall components, and require specific methods for their release and simultaneously minimizing contaminations with intracellular proteins [19]. Destructive procedures are commonly performed for the extraction of AMPs from ground kernels [20], whereas from leaf tissue proteins can also be released using a non-destructive vacuum infiltrations, in which AMPs are washed out of the apoplast with low intracellular contamination [21].

Due to their small size, AMPs are commonly overlooked and underrepresented in genome annotations of plants [22–24]. Similarly, AMPs are also underrepresented in conventional, gel-based proteome studies, due to difficulties in detecting basic peptides with high p*I* level and small molecular sizes (<10 kDa) [25]. Small cysteine-rich peptides are not amenable for most methods routinely used for large proteins and even AMPs that accumulate to high levels in transgenic plants have been shown to be barely detectable on immunoblots [3,26]. In the past, the production of efficient antibodies with affinity to the mature peptide has been shown to be problematic [3,27] and their small size does usually not allow for tagging without negatively influencing their *in vivo* activity and likely artificially enhancing their stability.

Recent progress and developments in mass spectrometry have expanded the field of proteomics from merely protein profiling to the accurate quantification of proteins. The shift from gel-based to gel-free shotgun proteomics allows for high throughput and label-free quantitative comparison of biological samples, opening new research possibilities in plant sciences [28–30]. Particular small, cysteine-rich peptides could benefit from this development, as these peculiar molecular features make them ineligible for most classical gel-based procedures. However, such high throughput methods for the analysis of multiple AMP families from plant tissue are lacking.

The wild tobacco (*Nicotiana attenuata*) has been widely used as an ecological model plant and for field studies of gene function. The development of a stable transformation procedure for this species [31] allowed for the manipulation of different layers of plant defenses and revealed genes important for defense against herbivores under natural field conditions [32]. We transformed wild tobacco plants with constructs for the ectopic expression of various AMPs to increase the plant’s resistance against microbes due to peptide accumulation in the apoplast. As *in planta* stability cannot be predicted, we chose 10 different AMPs for ectopic expression, including peptides from avian and amphibian origin (Table 1).

**Table 1:** **Acronyms of the transgenic** ***Nicotiana attenuata*** **lines and molecular properties of the ectopically expressed antimicrobial peptides**

**Plant line**	**Peptide name**	**Peptide family**	**Organism of origin**	**Monoisotopic mass [Da]**	**p** ***I***	**GenBank**
**DEF1**	NaDefensin1	defensin	*Nicotiana attenuata*	5475.68	9.33	[KF939593]
**DEF2**	NaDefensin2	defensin	*Nicotiana attenuata*	5300.58	9.08	[KF939594]
**VRD**	VrD1	defensin	*Vigna radiata*	5118.33	9.06	[AY437639]
**FAB**	Fabatin-1	defensin	*Vicia faba*	5236.40	9.12	[EU920043]
**ICE**	Mc-AMP1	knottin	*Mesembryanthemum crystallinum*	4213.92	9.30	[AF069321]
**PNA**	Pn-AMP2	hevein	*Ipomoea nil*	4179.68	8.52	[U40076]
**ESC**	Esculentin-1	esculentin	*Rana plancyi fukienensis*	4781.74	9.63	[AJ968397]
**SSP**	Spheniscin-2	avian defensin	*Aptenodytes patagonicus*	4504.29	11.63	[P83430]
**LEA**	LJAMP2	lipid-transfer protein	*Leonurus japonicus*	9119.53	9.02	[AY971513]
**CAP**	sheperin I +	glycine rich protein	*Capsella bursa-pastoris*	2360.95 +	7.28	[HQ698850]
				3257.29	7.28	
	sheperin II					

Here we describe the development of a peptide extraction method, capable of supporting high throughput plant screenings to confirm stable expression of a variety of different AMPs (with molecular masses ranging from 2.3 to 9.1 kDa and isoelectric points between 7.3 and 11.6). Our goal was to develop a method that allows for the rapid processing of many samples with relatively small volumes without requiring complex purification or chromatographic steps. The direct analysis of the intercellular fluid by nanoUPLC-MS^E^ allows for the (qualitative) detection of extracellular AMP deposition and even the (quantitative) comparison of peptide amounts among the different transgenic plant lines. Furthermore, this method does not rely on the availability of antibodies and can be easily adapted to other plant species or could be used to analyze endogenous AMP levels.

## Results

### Ectopic expression of AMPs in transgenic *N. attenuata* plants

**Figure 1:**
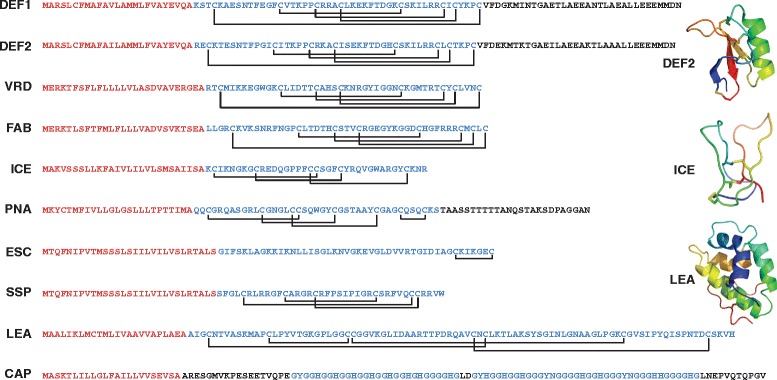
**Acronyms of the transgenic** ***N. attenuata*** **lines and the amino acid sequences of the ectopically expressed antimicrobial peptides (AMPs).** The N-terminal signal peptides are indicated in red, the mature peptide sequences are shown in blue and C-terminal or other domains in black. Cysteine residues which are connected by disulfide bridges are indicated. The simulated 3D structures of the DEF2, LEA and ICE peptides were retrieved from SWISS-MODEL (http://swissmodel.expasy.org/) and drawn with PYMOL softwarepackage 0.99rc6 (2006 DeLano Scientific).

For the ectopic expression of AMPs in the wild tobacco (*N. attenuata*), ten different transformation constructs harboring ten different antimicrobial peptides (AMPs) were constructed. Two of the peptides (DEF1 and DEF2) were endogenous AMPs from *N. attenuata* and were ectopically expressed in all plant tissues. Most of the other peptides were derived from plants (see Table 1) and selected to span the range of diversity found in the various AMP families (e.g. defensins, heveins, knottins, lipid-transfer proteins and glycin-rich peptides). Additionally, two animal peptides (from frog and penguin) were tested for their suitability to be expressed in *N. attenuata*. The stable transformation of *N. attenuata* was performed by *Agrobacterium* mediated gene transfer [31] and all peptides were expressed under the control of a constitutive 35S promoter. To direct their channeling into the protein secretion pathway, all peptides contained their native N-terminal signal peptide (Figure 1). Only the animal derived ESC and SSP constructs were fused to a plant signal peptide of the polygalacturonase-inhibiting protein (PGIP) leader sequence from *Phaseolus vulgaris*, which has been shown to target peptides for secretion in *N. tabacum* [33]. The complete sequences of the pre-peptides and the composition of the disulfide bridges from all AMPs are illustrated in Figure 1. Due to inconsistent naming of the peptides in the literature we use the acronyms of the plant lines from Table 1 also as a synonym for the peptides or the peptide genes. All transformed plants were thoroughly screened following the optimized protocol described in Gase *et al.* [34] to find homozygous, single copy lines with stable transgene expression confirmed by qRT-PCR and excluding epigenetically silenced plant lines [35]. Although gene expression analysis confirms the functional expression of a transgene, it provides no information about actual protein levels or stability of the ectopically expressed peptide within a plant. Therefore we extend the screening procedure with a method that allows for the comparison of peptide abundances.

### Selective peptide isolation by intercellular fluid extraction

**Figure 2:**
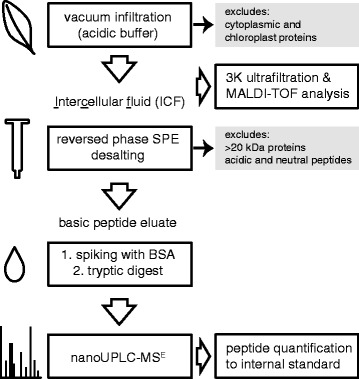
**Schematic representation of the workflow used for sample preparation of antimicrobial peptides (AMPs).** Intercellular fluid (ICF) was extracted by vacuum infiltration and desalted using reversed phase solid phase extraction cartridges (SPE). The samples were spiked with bovine serum albumin (BSA) which served as internal standard, tryptically digested and analyzed by nanoUPLC-MS^E^. Final peptide quantity was calculated and expressed as pmol per g fresh mass (FM).

The subcellular localization of the AMPs requires specific methods for a selective extraction. We modified a vacuum infiltration/centrifugation protocol [36] for the extraction of the apoplastic or intercellular fluid (ICF) from *N. attenuata* leaves (Additional file 1). ICF samples should theoretically contain only proteins and peptides from the apoplast and loosely bound cell wall proteins, as the cytoplasmic membrane remains undamaged during processing. To specifically enhance the solubility of basic peptides we used two different infiltration buffers, both containing high concentrations of salt and both with acidic pH (MES buffer pH 5.5 and citric acid buffer pH 3.0). The infiltration of about 5–6 leaves per plant allowed the recovery of 2.5–3 mL yellowish ICF. The overall yield among all plants was relatively homogenous with a mean value of 320 μL ICF per g fresh mass (FM) (±30 μL, n = 33 plants). By using a gentle centrifugation force (300 × g) tissue damage and intracellular protein contamination could be avoided, which would be indicated by a greenish color of the ICF. For all downstream MS based applications a rigorous desalting of the ICF samples was necessary. We initially used small volume (500 μL) ultrafiltration devices with a 3 kDa cut-off and analyzed samples by MALDI-TOF mass spectrometry (Figure 2). To also target extremely small <3 kDa peptides and simultaneously exclude >20 kDa proteins, we switched to reversed phase SPE cartridges for desalting and used a three-step elution to sequentially elute peptides by their charge for a higher purification and enrichment of basic peptides (Figure 2). With this procedure small volume samples could be rapidly desalted, reduced in sample complexity and enriched for AMPs and allowed the processing of multiple samples in parallel for nanoUPLC-MS^E^ analysis.

### AMP mass mapping by MALDI-TOF mass spectrometry

**Figure 3:**
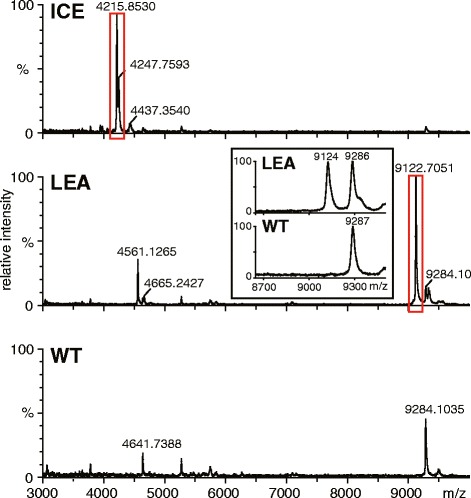
**Comparison of the MALDI-TOF mass spectra acquired from the intercellular fluid of WT and transgenic ICE and LEA lines.** ICF was extracted with citrate buffer (pH 3.0), desalted by ultrafiltration (VWR 3K columns) and analyzed in linear ion mode in the mass range 1–10 kDa. Peaks within the mass ranges of the expressed peptides are highlighted. The inset shows the MALDI-TOF MS analysis of the supernatant from WT and LEA lines (35 mL concentrated by Amicon 3K columns).

For an initial comparison of the peptide mass pattern of transgenic with those of WT plants, the desalted crude ICF extracts were subjected to analysis by Matrix-Assisted Laser Desorption/Ionization – Time-of-Flight Mass Spectrometry (MALDI-TOF MS). This approach was chosen as it is well suited for the rapid screening of peptide samples of low complexity due to its simplicity. Samples were analyzed in linear ion mode in the *m/z* range of 1,000–10,000 to cover the expected masses of all peptides (2.3 to 9.1 kDa). Only in two of the transgenic lines, we found a peak within the expected mass range of the expressed peptides for ICE – 4,215.85 Da (calculated monoisotopic mass 4,213.92 Da) and LEA – 9,122.71 Da (calculated monoisotopic mass 9,119.53 Da) (Figure 3). This was a strong indication for AMP accumulation and successful localization within the apoplast. The peak masses indicated full mature peptide length without truncations or proteolytic loss. However, with this method we found no evidence of peptide accumulation for most of the other transgenic lines, regardless of type of ultrafiltration device used (Additional file 2). To test for an eventual leakage of the peptides during ICF processing, we also concentrated and analyzed the used infiltration buffer (hereafter called supernatant) which remains after leaf removal following the vacuum infiltration (Additional file 1). Even the analysis of the supernatant revealed a peak for the LEA line, indicating the partial release of this peptide into the supernatant during the vacuum infiltration process (Figure 3, inset).

### AMP identification by nanoUPLC–MS^E^

To confirm AMP accumulation on the sequence level, ICF samples were tryptically digested and the obtained peptides were separated by nanoflow ultra-performance chromatography (nanoUPLC) for the detection by tandem mass spectrometry using MS^E^ analysis known as data-independent acquisition (DIA) [37]. The chosen mass spectrometric approach relies on the acquisition of alternating low/high collision energy data. The high sampling rate in MS^E^ data acquisition enables collection of sufficient data points to quantify peak ion intensities and was implemented in the label-free quantification of proteins based on observation that the intensity of three most intense (most efficiently ionized) tryptic peptides (Hi3 method) of a protein can be used as a measure of its abundance [38]. For nanoUPLC-MS^E^ analysis, ICF samples were desalted by reversed phase SPE according to our flowchart (Figure 2) and 5 μL of the final eluted fraction was spiked with 1 pmol bovine serum albumin (BSA), followed by digestion with trypsin. Since BSA does not occur in plants, it could function as an internal standard for quantification. To assess the applied quantification method, linear response and analytical reproducibility were considered. To this end serial dilutions were injected, corresponding to 2.5-25 μL ICF sample containing BSA amounts ranging from 50-500 fmol.

Among all identified tryptic peptides several could be reliably matched to the sequences of the overexpressed AMPs (Table 2). As most of the expressed AMPs do not naturally occur in *N. attenuata*, the appearance within the transgenic plants could confirm AMP expression, not only for the ICE and LEA lines, but also for the DEF1, DEF2, VRD, FAB and PNA genotypes. With this method overall 7 of 10 *N. attenuata* genotypes could be tested positive regarding peptide expression and showed peptide secretion into the apoplast. From the lipid-transfer protein of the LEA line up to 7 tryptic peptides could be identified, resembling 88% of the mature peptide sequence. Although most AMPs result only in a small number of tryptic peptides (Additional file 3), due to their small sizes, the sum of all detectable peptides resulted in more than 50% sequence coverage (except FAB, with only 34%) (Table 2). In comparison, from the internal standard (BSA) up to 34 tryptic peptides could be recovered resembling 59.8% sequence coverage. All tryptic peptides were unique and could unmistakably be matched to the respective AMPs. The defined amount of BSA spiked into the samples, allowed for the calculation of the molar concentration of each AMP per mL ICF or per g fresh mass (FM), based on the comparison of the internal standard to the peptides of interest [38]. In this way the absolute abundance of a peptide could be calculated for each sample.

**Table 2:** **Tryptic peptides of overexpressed AMPs detected by nanoUPLC-MS** ^**E**^ **in the intercellular fluid of** ***N. attenuata*** **plants**

**Line**	**Pep score**	**Calc.**	**Exp.**	**Rt [min]**	**Δ ppm**	**Sequence**	**Sequence coverage**
		**[MH]** ^**+**^	**[MH]** ^**+**^				
**DEF1**	8.41	1999.9077	1999.9001	36.36	3.77	AESNTFEGFC*VTKPPC*R	35.4%
	8.08	1000.4028	1000.4050	24.83	−2.21	C*IC*YKPC*	14.6%
**DEF2**	8.87	1977.9619	1977.9522	39.11	4.93	TESNTFPGIC*ITKPPC*R	36.2%
	7.39	707.3444	707.3393	36.28	7.17	AC*ISEK	12.8%
	7.79	938.3905	938.3894	19.83	1.24	C*LC*TKPC*	14.9%
**VRD**	8.49	1465.6243	1465.6233	25.76	0.65	C*LIDTTC*AHSC*K	26.1%
	8.44	1089.4137	1089.4163	33.56	−2.42	TC*YC*LVNC*	17.4%
	7.03	1534.6249	1534.6270	23.58	−1.41	GMTRTC*YC*LVNC*	26.1%
**LEA**	10.27	1518.7911	1518.7911	39.09	0.01	SYSGINLGNAAGLPGK	17.6%
	9.91	1925.8779	1925.8732	38.39	2.39	C*GVSIPYQISPNTDC*SK	18.7%
	8.35	1236.6117	1236.6116	37.25	0.14	MAPC*LPYVTGK	12.1%
	9.32	1061.4906	1061.4867	28.46	3.66	GPLGGC*C*GGVK	12.1%
	9.64	1020.5135	1020.5143	20.98	−0.74	AIGC*NTVASK	11.0%
	9.52	992.4647	992.4653	22.49	−0.56	QAVC*NC*LK	8.8%
	9.16	715.4095	715.4097	28.81	−0.31	GLIDAAR	7.7%
**PNA**	6.37	3421.3268	3421.3042	37.93	6.60	LC*GNGLC*C*SQWGYC*GSTAAYC*GAGC*QSQC*K	73.2%
**FAB**	7.58	1924.8133	1924.8100	31.21	1.74	FNGPC*LTDTHC*STVC*R	34.0%
**ICE**	9.26	1879.7229	1879.7198	39.08	1.68	EDQGPPFC*C*SGFC*YR	40.5%
	8.62	716.3829	716.3838	25.60	−1.27	QVGWAR	16.2%
	7.25	2252.8720	2252.8730	43.86	−0.43	GC*REDQGPPFC*C*SGFC*YR	48.6%

Carbamidomethylated cysteine indicated as C*; Δ ppm = 10^6^(M_tn_ − M_exp_)M_tn_^−1^.

### AMP quantification by nanoUPLC–MS^E^

**Figure 4:**
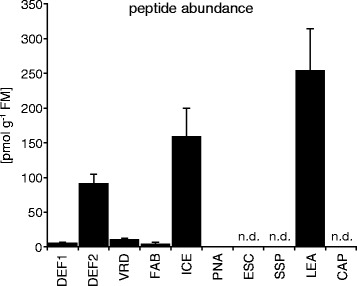
**Comparison of peptide abundance calculated from LC-MS** ^**E**^ **data of different transgenic** ***N. attenuata*** **lines.** Intercellular fluid (ICF) was extracted with MES buffer (pH 5.5) and desalted using reversed phase cartridges. The samples were analyzed by nanoUPLC-MS^E^ and the peptide abundance calculated based on the relation between the averages of the intensity of the three most intense peptides of the internal standard (BSA) to the peptides of interest [38]. Peptide abundances are shown as pmol per g fresh mass (FM) ± SEM from 3 biological replicates per genotype (6 biological replicates for DEF2, ICE and LEA lines); n.d. = not detected.

**Figure 5:**
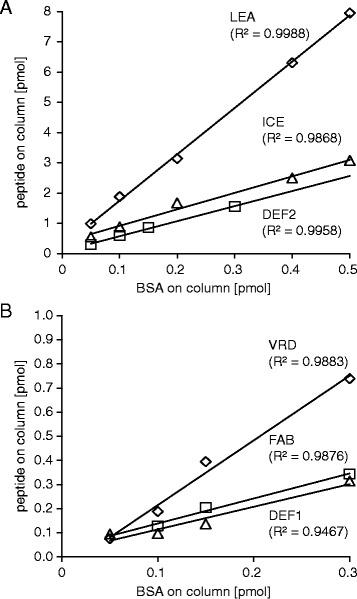
**Linear dynamic range of nanoUPLC − MS** ^**E**^ **measurements of AMPs.** To determine the linear dynamic range of quantification, the calculated peptide amounts [fmol/column] from 3–5 technical replicates were plotted against the corresponding amount of BSA in the sample (50–500 fmol); BSA was linear in the full range tested. **(A)** Linear regression (R^2^) shown for the high abundant AMPs (LEA, ICE and DEF2). **(B)** Linear regression (R^2^) shown for the low abundant AMPs (VRD, FAB and DEF1).

Although peptide abundance could be confirmed for the PNA, FAB, DEF1 and VRD lines, the quantitative comparison indicated relatively low peptide amounts within these lines with 0.2–11 pmol g^−1^ FM (Figure 4). In particular the PNA peptide was very low abundant and on the limit of detection since it could only be detected in 1 out of 3 biological replicates. In contrast, the DEF2, ICE and LEA lines indicated very high peptide amounts with 92–254 pmol g^−1^ FM (Figure 4). This confirmed the desired high extracellular peptide accumulation within the apoplast, as it would be required for these transgenic plants. To estimate the accuracy of the quantification method, the linear response of AMPs to the internal standard BSA (which was assessed for linear responses within the used concentrations) was determined by analyzing serially diluted samples. For the high abundant peptides (Figure 5A) as well as the low abundant peptides (Figure 5B) the MS^E^ based quantification revealed a wide linear dynamic range among the injected concentrations, which reached for the LEA peptide up to 8000 fmol. Since we worked with native concentrations from biological samples we could not further exceed these values to reach possible saturation limits. To confirm repeatability of the quantitative results we analyzed 3 additional replicates from the plant lines with high peptide abundance (DEF2, ICE and LEA). For all 6 biological replicates a high AMP accumulation could be confirmed and showed among all individual quantifications a small technical error (Additional file 4). The averaged relative standard deviation (standard deviation of each technical replicate divided by its mean and multiplied by 100) was 21.1% for all the measured peptides and best for the LEA peptide with only 11.0%.

**Figure 6:**
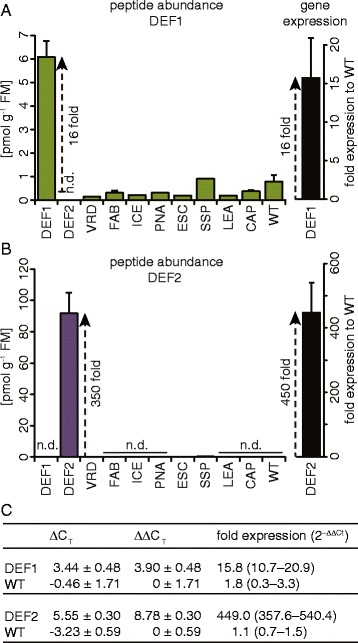
**Comparison of endogenous DEF1 and DEF2 peptide abundance with strength of gene expression. (A)** The DEF1 overexpressing lines showed about 16-fold higher peptide amounts compared to the average found in all other lines. **(B)** The DEF2 overexpressing lines showed about 350-fold higher amounts compared to the average found in all other lines. **(C)** Calculation of fold differences in gene expression compared to WT using the comparative C_T_ method (ΔC_T_ = actin - defensin; ΔΔC_T_ = line - WT) with actin as reference gene (± SD, n = 4 plants).

As the DEF1 and DEF2 peptides were endogenous defensins of *N. attenuata,* peptide levels can be directly compared to native levels within untransformed WT plants. The DEF1 peptide could indeed be detected in the ICF of WT, as well as most other transgenic plants (Figure 6A). The DEF1 over-expression line showed the highest peptide amounts, which was about 16-fold higher than the average found in all other lines. This correlated with the expectations from gene expression data, where these lines showed on average a 16-fold increase in transcript level compared to WT. The DEF2 plants showed much higher transcript levels, which were on average 450-fold higher compared to WT (Figure 6B). This was as well consistent with the observed peptide amounts, which were 350-fold elevated compared to the basal amount found in some transgenic lines.

### ICF sample composition and protein localization

To illustrate general differences in protein composition of ICF extracts to total leaf extracts, we compared raw ICF samples (without SPE processing) with total soluble leaf proteins by SDS-PAGE (Additional file 5A). Both extraction methods showed distinct protein profiles. Very large proteins (>100 kDa) seem to be absent in the ICF samples whereas total soluble protein extracts were dominated by protein bands at around ~55 kDa and ~14 kDa which belong to the large (LSU) and small subunit (SSU) of ribulose-1,5-bisphosphate carboxylase (RuBisCO). The lack of these bands within the concentrated ICF samples indicates that these samples did not contain major intracellular contaminations and that cell lysis played only a minor role during the vacuum infiltration process. Furthermore we evaluated if the ICF samples were enriched in endogenous apoplastic peptides and performed database searches with the MS^E^ datasets. Since the abundance of non-target proteins was relatively low we used a 6 times higher concentration, than usually used for AMP quantification. Since the sample preparation method was specific for small cationic peptides (Additional file 5B), we commonly found endogenous AMPs within the ICF samples, belonging to the non-specific lipid-transfer protein (LTP), snakin or the plant defensin family (Additional file 5C). This shows that this method is suitable for the analysis of endogenous AMPs which are expected to be present in apoplastic fractions. But we also observed peptides belonging to the RuBisCO SSU and plastocyanin within most samples, which are both chloroplast proteins and indicate contamination from intracellular pools. Still, in a quantitative comparison intracellular proteins showed only 10–20% the abundance levels of the low abundant AMPs (DEF1, FAB and VRD), whereas compared to the high abundant AMPs (DEF2, ICE and LEA) they were only 0.6–1.5% as abundant (Additional file 5C). Thus it is unlikely that the expressed AMPs merely leaked from intracellular pools.

As we had evidence of peptide release into the infiltration buffer during ICF processing we also analyzed the remaining supernatants after the extractions (Additional file 1). We concentrated 15 mL supernatant using SPE cartridges and analyzed 5% of the eluted fraction (equivalent to 750 μL supernatant). Most AMPs could be detected in the supernantant as well and the quantitative comparison revealed a similar pattern as observed from the ICF samples. The highest peptide amounts were found in the DEF2, ICE and LEA lines (Additional file 6) and smaller amounts found for the DEF1, FAB and VRD lines, indicating that peptides are released into the buffer nearly proportional to the overall peptide amount found in the apoplast.

## Discussion

The facile absolute quantification of plant proteins has the potential to substantially advance many research areas, however sample complexity still thwarts robust quantifications, particularly for cationic AMPs. In this study, we developed a high throughput method for extracting and processing intercellular fluid from leaf tissue, generating samples suitable for mass spectrometric analysis and allowing the detection and quantification of different ectopically expressed AMPs in transgenic *N. attenuata* plants. We adapted a vacuum infiltration method for *N. attenuata* and tested different desalting procedures to analyze peptide abundances with nanoUPLC-MS^E^ in a high throughput fashion (Figure 2). As a result we could confirm the accumulation of heterologously expressed peptides within the apoplast and could quantify their abundance in comparison to endogenous AMPs.

### AMPs require specific extraction methods

Many purification methods make use of the unique biochemical properties of AMPs, such as their small size, their positive charge, their tolerance to acids and heat or even the presence of disulfide bridges, as done recently by Hussain *et al*. [39]. We took advantage of the subcellular localization within the apoplast and the selectivity of extraction during vacuum infiltration. The obtained intercellular fluid (ICF), also commonly called apoplastic wash fluid (AWF) or intercellular washing fluid (IWF), shows a tremendously reduced complexity compared to crude, whole cell fractions, containing cytoplasmic and chloroplast proteins. Particular dominant proteins of the photosystem (RuBisCO) were strongly reduced in the ICF extracts (Additional file 5) similar as shown in Delannoy *et al*. [9]. To achieve an optimized infiltration process, the ICF extraction protocol needs to be adapted to each plant species [40]. The salt concentrations and the pH of the infiltration buffer also have a large influence on the protein extraction efficiency [41]. In general, mild acids are commonly used for the extraction of AMPs as shown for the isolation of floral defensins from the ornamental tobacco, *N. alata* [27]. In addition, has the use of acidic buffers the advantage of reducing phenolic browning of the extracts, which is a common problem for other protein extraction buffers used for *N. attenuata* and other tobacco species, e.g. for trypsin protease inhibitor extraction [42]. For the selective enrichment of AMPs we tested the pre-cleaning of large proteins with a 30K cut-off ultrafiltration step or heat clearance prior to desalting (10 min at 80°C) and could confirm the heat stability of the ICE and LEA peptides. But we generally omitted these steps as they did not improve the overall sample quality, in fact the manufacturer and type of the ultrafiltration device had rather a strong influence on ICF sample composition (Additional file 2). Ultrafiltration can separate proteins only by size, but allows no further purification. Desalting with reversed phase SPE cartridges allowed not only size exclusion, but also separation by charge, which could remove contaminants (Additional file 5B). As the sequentially elution steps during SPE processing resulted in a further reduction of the ICF sample complexity and could enrich basic peptides in the final fraction, it was the preferred method for all nanoUPLC-MS^E^ measurements. The whole method was developed as a universal extraction and purification of cationic peptides, and has been also proven to be useful for the extraction of endogenous AMPs. Since the method was stringent for cationic peptides, not many other proteins could be found within these samples and the degree of intracellular contamination was overall very low. Only intracellular proteins <20 kDa (e.g. RuBisCO small subunit and plastocyanin) could co-elute and were commonly observed in most SPE desalted samples, whereas parts of the RuBisCO large subunit could only be detected in about half of the samples (Additional file 5). Considering that proteins from the photosystem are the most abundant proteins in plants, the up to 2 orders of magnitude higher concentrations of the overexpressed AMPs show that intracellular contamination was basically negligible. Since there is no all-round method which could cover conditions of all AMPs, it was not surprising that the method was not optimal for the CAP peptides. These glycine-rich peptides were not cleavable by trypsin and likely need specific modifications regarding the desalting process or the use of different digestion enzyme to increase the chances of later detection.

### NanoUPLC-MS^E^ based AMP quantification

Although AMPs have been expressed in various plant species there have rarely been attempts to quantify AMP accumulation in transgenic plants. *In vitro* test have shown potential for the use of RP-HPLC and NMR based methods, but only for the quantification of pure fractions of cyclotides, and showed limitations for spectrophotometric methods for these peptides [43]. For the direct analysis of cyclotides from plant extracts even MALDI-TOF MS based quantitative methods have been developed [44]. We used MALDI-TOF analysis for peptide mapping and could only detect two very abundant peptides, probably due to the limited resolution and sensitivity of this method for peptides at molecular masses above 3 kDa. Furthermore one of the biggest disadvantages is the lack of sequence information. Through technical advances in high-performance LC separation of peptides and development of modern mass spectrometer with high resolution and scanning rates, label-free quantification of proteins has been implemented in proteomic routine [45,46]. This simple and cost-efficient method enables simultaneous protein quantification across many samples without tedious protein or peptide derivatization. Hi3 nanoUPLC-MS^E^ based quantification of proteins, used in this study, combined advantages of ultra-performance liquid chromatography that provides high reproducibility in nanoUPLC runs with high sampling rate of MS^E^ data acquisition required for accurate quantitative analysis [30]. Instead of analyzing secreted proteins from cell culture media [47,48], we injected desalted and tryptically digested ICF samples derived from plant tissue for a direct quantification.

Despite the achieved *in vitro* precisions, variability among samples prepared from complex tissues is the major limitation in the application of quantitative proteomics [38,49], which is particularly true for cell wall bound peptides. Despite the variability among biological replicates resulting from separate infiltration procedures (Additional file 4), we found consistent patterns of peptide abundance and, among the highly abundant peptides, a remarkable large linear dynamic range (LEA peptide showed R^2^ > 0.998 for up to 8000 fmol). It should be noted that the small size of most AMPs strongly limits the options in selecting best ionizable tryptic peptides for quantification measures [38], in contrast to very large and abundant plant proteins, which yield a much broader variety of tryptic peptides and allow more precision in quantification [37]. When necessary, we also included miss-cleaved tryptic peptides to be able to perform the Hi3 peptide quantification for all AMPs. This was the most appropriate method as it resulted in good linear ranges for most AMPs compared to BSA. But the defensins (DEF1, DEF2 and VRD) would show a higher linearity if the sum of intensity of all matched peptides would be used for quantification. However, as this procedure decreased accuracy for the LEA and ICE peptides, we used the Hi3 method for quantification of all peptides to maintain comparability among all the different AMPs. Another possible way improving further accuracy could be achieved by using a peptide standard of a similar size as the AMPs.

### AMP localization and expression in plants

In the ornamental tobacco (*N. alata*) two floral defensins had been previously reported to be localized only in the vacuole, suggesting that their carboxyl-terminal pro-domains have a protein trafficking function [50,51]. The orthologous DEF2 peptide of *N. attenuata* has 100% amino acid similarity to *N. alata* NaD1 and we expected an accumulation within the vacuole. However, in transgenic *N. attenuata* plants ectopically expressing this peptide large amount was detectable within the ICF samples, consistent with their secretion into the apoplast (Figure 4). Although the DEF1 peptide shared 86% protein sequence similarity with DEF2, their expression strength and the amount of accumulated peptide differed dramatically between these lines. DEF2 was much more over-expressed than DEF1, an observation that strongly calls into question the ability to predict suitable candidates for over-expression studies based merely on sequence data. The overall tremendous differences in AMP accumulation amongst all plant lines emphasize the value of a direct assessment of peptide amounts. In fact, the PNA and ESC lines were initially among our most promising candidates, as for these peptides a successful expression has been reported in *N. tabacum* [33,52]. But the extreme low detectability and the C-terminal pro-domain of the PNA peptide are indicators that this peptide might be intracellular localized, whereas the amphibian esculentin-1 peptide was undetectable in the ESC line and has been reported to show signs of degradation by exopeptidases in *N. tabacum* [33]. However, the lack of AMP detectability could either indicate instability or amounts below the detection limit, both valuable reasons to exclude the plant lines from further studies. AMPs usually need to accumulate to large amounts, as was found in the DEF2, ICE and LEA lines, to exert a biological function. Interestingly, most of the peptides could also be found within the supernatant, which remained after vacuum infiltration (Additional file 6). More strikingly, the overall pattern of peptide abundance was very similar among ICF and supernatant samples. This suggests that either the peptides readily diffuse out of the apoplast during the infiltration process, or were washed from the leaf surface. The analysis of a pure leaf surface wash would be a promising future experiment, which could further clarify this hypothesis. A leaf surface deposition by glandular trichomes is in particularly likely for the DEF1 and DEF2 peptides as the concentrations (per mL) were only 10–19 times lower in the supernatant than the concentrations (per mL) from the ICF samples. In contrast, the concentrations of the other peptides were 44–143 times lower in the supernatant. However, the active secretion of these peptides from the roots could not be confirmed. We harvested hydroponic solutions of the transgenic plants and concentrated it using SPE cartridges. From the eluted fractions 10% were analyzed (equivalent to 1.7 mL root exudate), showing no match for any of the expressed AMPs.

## Conclusions

Bio-analytical technology has recently made tremendous progress in the development of peptide quantification techniques and opens many opportunities for applications [30]. The analyses of peptide fluctuations within the plant cell wall, after wounding or infection, are possible examples. The most limiting factor for peptide quantification is perhaps the bias resulting from sampling and sample preparation. Accurate quantifications of absolute *in vivo* concentrations are challenging due to different chemical properties of different peptides which result in diverging affinities for extraction and/or purification. Further improvement is expected if digestion methods other than trypsin-assisted proteolysis will be tested for small polypeptides with a limited number of Lys and Arg in the chain. Here we show that a relatively simple extraction procedure can efficiently release a diverse set of antimicrobial peptides from leaf tissues to provide the basis for a universal method that achieves reliable peptide quantification results by nanoUPLC-MS^E^ that applies label-free quantification.

## Methods

### Construction of plant transformation vectors

The sequences of different genes coding for antimicrobial peptides were selected from the PhytAMP database (http://phytamp.pfba-lab-tun.org/main.php) and from NCBI (Table 1). The animal peptides SSP and ESC were fused to the signal peptide of the polygalacturonase-inhibiting protein (PGIP) leader sequence from *Phaseolus vulgaris* as described in [33]. All AMP sequences were tested for the presence of a signal peptide using the SignalP 3.0 Server (http://www.cbs.dtu.dk/services/SignalP/). The sequences for the SSP, ESC, PNA, VRD and FAB constructs were manually adapted to the codon usage table of *N. tabacum* (http://gcua.schoedl.de/). Genes from *N. attenuata* were directly PCR amplified from leaf cDNA and the CAP gene was amplified from root cDNA of a wild *Capsella bursa-pastoris* plant collected in front of the Institute for Chemical Ecology. Most other constructs were synthesized in sequential PCR reactions with overlapping 40 bp primers and did not require the availability of cDNA from the organism of origin. All genes were cloned in pSOL9 binary plant transformation vectors under a constitutive cauliflower mosaic virus promoter (35S) described in Gase *et al.* [34]. Two peptides had amino acid substitutions compared to their native sequence DEF2 (Ile102Met) and Esc (Met28Leu).

### Plant transformation and growth conditions

*Nicotiana attenuata* Torr. ex S. Watson seeds were originally collected in 1988 from a natural population at the DI Ranch in Southwestern Utah. Wild-type seeds from the 30^th^ inbreed generation were used for the construction of transgenic plants and as WT controls in all experiments. Plant transformation was performed by *Agrobacterium tumefaciens*-mediated gene transfer as previously described [31]. Transgenic plant lines were screened as described in Gase *et al.* [34] and Weinhold *et al.* [35]. Homozygous, single insertion T_3_ plant lines used in MS^E^ quantification were: LEA 1.7.1 (A-09-721), PNA 8.6.1 (A-09-823), FAB 9.3.1 (A-09-865), ICE 6.4.2 (A-09-748), CAP 6.4.1 (A-09-949), DEF1 F.3.1 (A-09-167), DEF2 C.7.1 (A-09-230), SSP 6.5.1 (A-09-671), ESC 1.3.1 (A-09-693) and VRD 4.7.1 (A-09-668). Additional lines used for MALDI analysis were: ICE 1.1.9 (A-09-653), SSP 4.6.1 (A-09-775), ESC 2.7.1 (A-09-778) and VRD 1.9.1 (A-09-652). Seeds were germinated as described in Krügel *et al.* [31] and incubated in a growth chamber (Percival, day 16 h 26°C, night 8 h 24°C). Ten-days-old seedlings were transferred to communal Teku pots and ten days later into individual 1L pots and cultivated in the glasshouse under constant temperature and light conditions (day 16 h 26–28°C, night 8 h 22–24°C). For the collection of root exudates, plants were grown in hydroponic culture in individual 1L pots containing 0.292 g/L Peter’s Hydrosol (Everri, Geldermalsen, the Netherlands). After 25 days of growth the hydroponic solution from 5 plants was pooled and 50 mL sterile filtered using a Minisart sterile filter 0.2 μm (Sartorius). The solution was concentrated using reversed phase SPE cartridges (see below).

### Vacuum infiltration and peptide extraction

The Intercellular fluid (ICF) was extracted from 35–45 days old *N. attenuata* plants using a modified vacuum infiltration method [36]. Per plant 5–6 fully expanded leaves were detached and, if necessary, the midrib excised with a scissor (Additional file 1). The leaves were submerged in 40 mL chilled (4°C) infiltration buffer, either MES buffer pH 5.5 (20 mM MES/KOH pH 5.5, 1M NaCl, 200 mM KCl, 1 mM thiourea) or a citrate buffer pH 3.0 (20 mM citric acid/sodium citrate pH 3.0, 200 mM CaCl_2_, 1 mM thiourea). The submerged leaves were placed into a desiccator and a vacuum of -80 kPa applied for 5 minutes. Air bubbles were dislodged with gentle agitation and the apoplastic spaces were filled with infiltration buffer by slowly releasing the vacuum, indicated by darkening of the leaves. Infiltrated leaves were surface dried using paper towels and placed into a barrel of a 20 mL syringe, stuffed with glass wool at the tip and hung in a 50 mL centrifuge tube. ICF was released by slow centrifugation (300 × g) in a swing bucket rotor for 15 min at 4°C. The used infiltration buffer was clarified by centrifugation (20 min at 400 g) and 15 mL saved as “supernatant” (Additional file 1). Samples were frozen at -20°C until further processing.

### Peptide desalting

The peptide fractions of the ICF samples were desalted and concentrated either by ultrafiltration or reversed phase SPE cartridges. Prior ultrafiltration some ICF samples were heat cleared at 80°C for 10 min in a heating block and the heat sensitive proteins removed by centrifugation in a table top centrifuge (16,000 × g, 10 min). The supernatant was desalted and concentrated with either Amicon Ultra-0.5 columns (Ultracel 3K Membrane) or with VWR Centrifugal Filters (modified PES 3K), both with a loading capacity of 500 μL and a 3 kDa size cut-off. Samples were re-loaded and centrifuged for 15 min at 14,000 × g at room temperature in a table top centrifuge, washed 3× with 450 μL Milli-Q water. Solid phase extraction was performed using Phenomenex Strata™ X 33 μm Polymeric Reversed Phase columns (30 mg/mL) as suggested by the manufacturer, conditioned prior use with 1 mL acetonitrile (ACN) and equilibrated with 1 mL Milli-Q water. From each sample 1 mL was consecutively applied until the whole sample was loaded. The column was washed 3× with 1 mL Milli-Q water. Elution was performed in three steps, eluting first the acidic peptides in 500 μL 40% ACN/water (v/v), second the neutral peptides in 500 μL 70% ACN/water (v/v) and finally the basic peptides in 500 μL 70% ACN/0.3% formic acid (v/v). AMPs were only detected in the final fraction. Samples were stored in the freezer at -20°C until further analysis.

### Matrix-Assisted Laser Desorption/Ionization Time-of-Flight (MALDI-TOF) mass spectrometry

Crude samples desalted by ultrafiltration were analyzed using a MALDI Micro MX mass spectrometer (Waters). All measurements were performed in the *m/z* range of 1,000–10,000 in linear ion mode. The lyophilized samples were reconstituted in 10 μL aqueous 0.1% TFA. One μL of sample was mixed with 1 μL aliquot of α-cyano-4-hydroxycinnamic acid (α-matrix, 10 mg/mL in ethanol/ACN, 1:1, v/v), and 1 μL of the solution was spotted onto a metal 96-spot MALDI target plate. The instrument was operated in positive ion mode, with 3.5 kV set on the sample plate, and 12 kV on the extraction grid. A nitrogen laser (337 nm, 5 Hz) was used for ionization/desorption and the extraction of ions was delayed by 500 ns. The pulse voltage was 1100 V and the detector voltage was set to 2.15 kV. MassLynx v4.1 software was used for data acquisition (Waters). Each spectrum was combined from 15 laser pulses. Angiotensin II, bradykinin, ACTH, insulin, cytochrome C, and myoglobin (all Sigma) at 1 to 10 pmol on target were used to calibrate the mass spectrometer.

### Sample preparation for nanoUPLC − MS^E^ analysis

Following SPE, 5 μL per sample were vacuum-dried for AMP quantification and 30 μL for non-target protein quantification (up to 50 μL were tested) and reconstituted in 50 μL of 50 mM ammonium bicarbonate buffer containing 1 pmol BSA (Sigma-Aldrich, purity ≥98%) used as internal standard. The proteins were reduced by addition of DTT to a final concentration of 10 mM, incubated for 30 min at 60°C and alkylated with 15 mM iodoacetamide in the dark for 30 min at room temperature. Proteolysis was carried out by adding 100 ng of sequencing grade porcine trypsin (Promega) at 37°C overnight. The samples were vacuum-dried and kept at -20°C. Prior analysis, the samples were re-dissolved in 20 μL 3% ACN/0.1% formic acid (v/v) solution.

### NanoUPLC-MS^E^

The peptide amounts were quantified using a nanoAcquity UPLC system on-line connected to a Q-ToF Synapt HDMS mass spectrometer (Waters). To test linearity to the internal standard 1 to 10 μL of the samples (10 – 100% sample loop volume) were injected containing final concentrations of BSA ranging from 50 – 500 fmol (on column). To estimate the biological and analytical reproducibility of the method 3-5 technical replicates were measured from each of the 3-6 biological replicates per genotype. Samples were concentrated on a Symmetry C18 trap-column (20 × 0.18 mm, 5 μm particle size, Waters) at a flow rate of 15 μL/min. The trap-column was on-line connected to a nanoAcquity C18 analytical column (200 mm × 75 μm ID, C18 BEH 130 material, 1.7 μm particle size, Waters) and the peptides were separated at a flow rate of 350 nL/min using following LC-gradient: 1 – 30% B (13 min), 30 – 50% B (5 min), 50 – 95% B (5 min), 95% B (4 min), 95% – 1% B (1 min) [Solvent (A): 0.1% formic acid in ultra-pure water; solvent (B) 0.1% formic acid in 100% ACN]. The eluted peptides were transferred through a NanoLockSpray ion source into the mass spectrometer operated in V-mode at a resolution of at least 10 000 (FWHM). LC-MS data were acquired under data-independent acquisition at constant collision energy of 4 eV in low energy (MS) mode, ramped in elevated energy (MS^E^) mode from 15 to 40 eV. The mass range (*m/z*) for both scans was 50–1,900 Da. The scan time was set at 1 sec for both modes of acquisition with an inter-scan delay of 0.2 sec. A reference compound, human Glu-Fibrinopeptide B [650 fmol/mL in 0.1% formic acid/ACN (v/v, 1:1)], was infused through a reference sprayer at 30 s intervals for external calibration. The data acquisition was controlled by MassLynx v4.1 software (Waters).

### Data processing and protein identification

The acquired continuum LC-MS^E^ data were processed using ProteinLynx Global Server (PLGS) version 2.5.2 (Waters) to generate product ion spectra for database searching according to Ion Accounting algorithm described by Li *et al.* [53]. The thresholds for low/ high energy scan ions and peptide intensity were set at 150, 30 and 750 counts, respectively. Database searches were carried out against Swissprot database (downloaded on Juli 27, 2011 http://www.uniprot.org/) combined with the known protein sequences of the AMPs at a False Discovery Rate (FDR) of 2%, following searching parameters were applied for the minimum numbers of: product ion matches per peptide (3), product ion matches per protein (5), peptide matches (1), and maximum number of missed tryptic cleavage sites (1). Searches were restricted to tryptic peptides with a fixed carbamidomethyl modification for Cys residues. For the quantification we used the Hi3 method, whereas a universal response factor was calculated from BSA (the averaged intensity of the three most intense peptides) compared to the intensity of the peptides of interest as described by [38].

### Total leaf extract and gel electrophoresis

For the comparison of the raw ICF protein composition with total leaf proteins intact leaves were ground in liquid nitrogen and 150 mg used for the extraction of total soluble proteins similar as described in Jongsma *et al.* [42]. ICF and total protein samples were desalted and concentrated by ultrafiltration (Amicon Ultra-0.5 3K). Protein concentrations were determined by the method of Bradford and 20 μg (respective 8 μg for ICF) separated by gel electrophoresis on a 8–16% Tris-Glycine Gel. Proteins and peptides were fixed in 5% glutaraldehyde and stained with coomassie brilliant blue.

### Gene expression analysis

The isolation of RNA and the qRT-PCR were performed as previously described [35] using the following primers: Def1-7F (5′- CGCTCCTTGTGCTTCATGG-3′), Def1-83R (5′- GTACTCTTAGCTTGCACCTCATAGGC-3′), Def2-21F (5′- CATGGCATTTGCTATCTTGGC-3′), Def2-98R (5′- TTGCTTTCTGTTTTGCATTCTCTAG-3′).

## Additional files

Additional file 1:
**Illustration of the vacuum infiltration procedure.** *N. attenuata* leaves were submerged in infiltration buffer and exposed to a vacuum inside a desiccator. A complete infiltration was indicated by the darkening of the leaves and a more translucent appearance. The remaining infiltration buffer was collected as “supernatant”. The infiltrated leaves were centrifuged and the extracted liquid was collected as intercellular fluid (ICF). Additional file 2:
**Comparison of MALDI-TOF mass spectra using different ultrafiltration devices.** Spectra were acquired from the intercellular fluid (ICF) of WT and transgenic plants in the mass range 1–10 kDa. Peaks within the expected mass ranges from the ICE and LEA lines are indicated. (A) ICF was extracted with citrate buffer (pH 3.0) and desalted by ultrafiltration (VWR 3K columns). (B) ICF was extracted with citrate buffer (pH 3.0), heat treated (80°C) and desalted by ultrafiltration (Amicon 3K columns). MALDI-TOF instrument was operated in linear ion mode. Additional file 3:
**Expected masses of AMP peptides after tryptic digest.** The peptides (minimum 300 Da) were computed using the Expasy server (http://web.expasy.org/peptide_mass/). Tryptic peptides confirmed by MS^E^ are underlined. Additional file 4:
**Biological and analytical variability of AMPs quantified using nanoUPLC-MS** ^**E**^ **.** The AMP abundance in 3–6 individual biological replicates is shown, each derived from the intercellular fluid extraction of a single *N. attenuata* plant. Error bars indicate the standard error of 3–5 technical replicates, n.d. = not detected. Additional file 5:
**ICF sample composition regarding non-target proteins and impurities.** (A) Comparison of the protein composition from total soluble plant extracts vs intercellular fluid (ICF) extracts. Dominant intracellular proteins such as the RuBisCO large subunit (LSU) and small subunit (SSU) are highlighted. ICF extracts (MES buffer pH 5.5) did not indicate the presence of major intracellular contaminations, but were enriched with the fraction of interest (peptides with a mass <10 kDa). (B) SPE peptide desalting removed larger proteins, impurities and enriched basic peptides. (C) Average abundance of the most commonly detected endogenous proteins calculated from LC-MS^E^ data of 11 different genotypes (n = 11, ± SEM) in relation to the AMPs of interest (shown in black). The spiked BSA standard (200 fmol on column) is shown in red. Commonly detected endogenous proteins were apoplastic AMPs (shown in yellow) and chloroplast proteins (shown in green). LTP = Lipid transfer protein. Additional file 6:
**Determination of AMP abundance in the supernatant.** (A) The supernatants after vacuum infiltration (MES, pH 5.5) were SPE desalted, spiked with BSA and analyzed using nanoUPLC-MS^E^, n.d. = not detected; (B) Comparison of all peptides from the supernatant of the respective genotype. (C) Comparison of DEF1 abundance in the supernatant. (D) Comparison of DEF2 abundance in the supernatant.

## Abbreviations

AMP: Antimicrobial peptide
ICF: Intercellular fluid
MS^E^: Elevated-energy mass spectrometry
