# Supplementary material for: Label-free nanoUPLC-MSE based quantification of antimicrobial peptides from the leaf apoplast of Nicotiana attenuata
Source: BMC Plant Biol. 2015 Jan 21;15:18. doi: 10.1186/s12870-014-0398-9 (PMC4318441; doi:10.1186/s12870-014-0398-9)
Supplement: Additional file 1: — Illustration of the vacuum infiltration procedure. N. attenuata leaves were submerged in infiltration buffer and exposed to a vacuum inside a desiccator. A complete infiltration was indicated by the darkening of the leaves and a more translucent appearance. The remaining infiltration buffer was collected as “supernatant”. The infiltrated leaves were centrifuged and the extracted liquid was collected as intercellular fluid (ICF). [file 12870_2014_398_MOESM1_ESM.pdf]

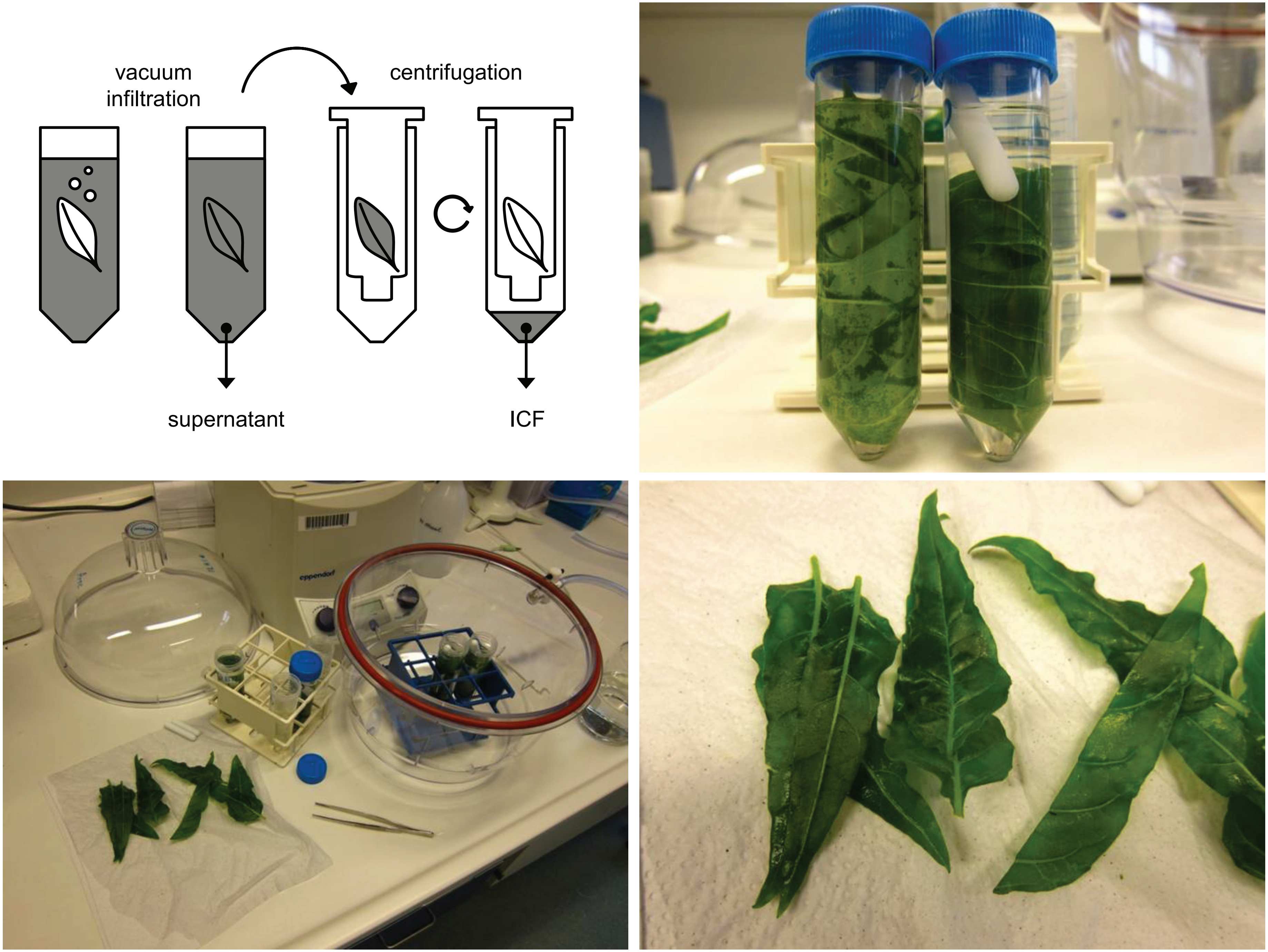

Additional file 1: Illustration of the vacuum infiltration procedure.
N. attenuata leaves were submerged in infiltration buffer and exposed to a vacuum inside a desiccator. A complete infiltration was indicated by the darkening of the leaves and a more translucent appearance. The remaining infiltration buffer was collected as “supernatant”. The infiltrated leaves were centrifuged and the extracted liquid was collected as intercellular fluid (ICF).
