# Supplementary material for: Label-free nanoUPLC-MSE based quantification of antimicrobial peptides from the leaf apoplast of Nicotiana attenuata
Source: BMC Plant Biol. 2015 Jan 21;15:18. doi: 10.1186/s12870-014-0398-9 (PMC4318441; doi:10.1186/s12870-014-0398-9)
Supplement: Additional file 2: — Comparison of MALDI-TOF mass spectra using different ultrafiltration devices. Spectra were acquired from the intercellular fluid (ICF) of WT and transgenic plants in the mass range 1–10 kDa. Peaks within the expected mass ranges from the ICE and LEA lines are indicated. (A) ICF was extracted with citrate buffer (pH 3.0) and desalted by ultrafiltration (VWR 3K columns). (B) ICF was extracted with citrate buffer (pH 3.0), heat treated (80°C) and desalted by ultrafiltration (Amicon 3K columns). MALDI-TOF instrument was operated in linear ion mode. [file 12870_2014_398_MOESM2_ESM.pdf]

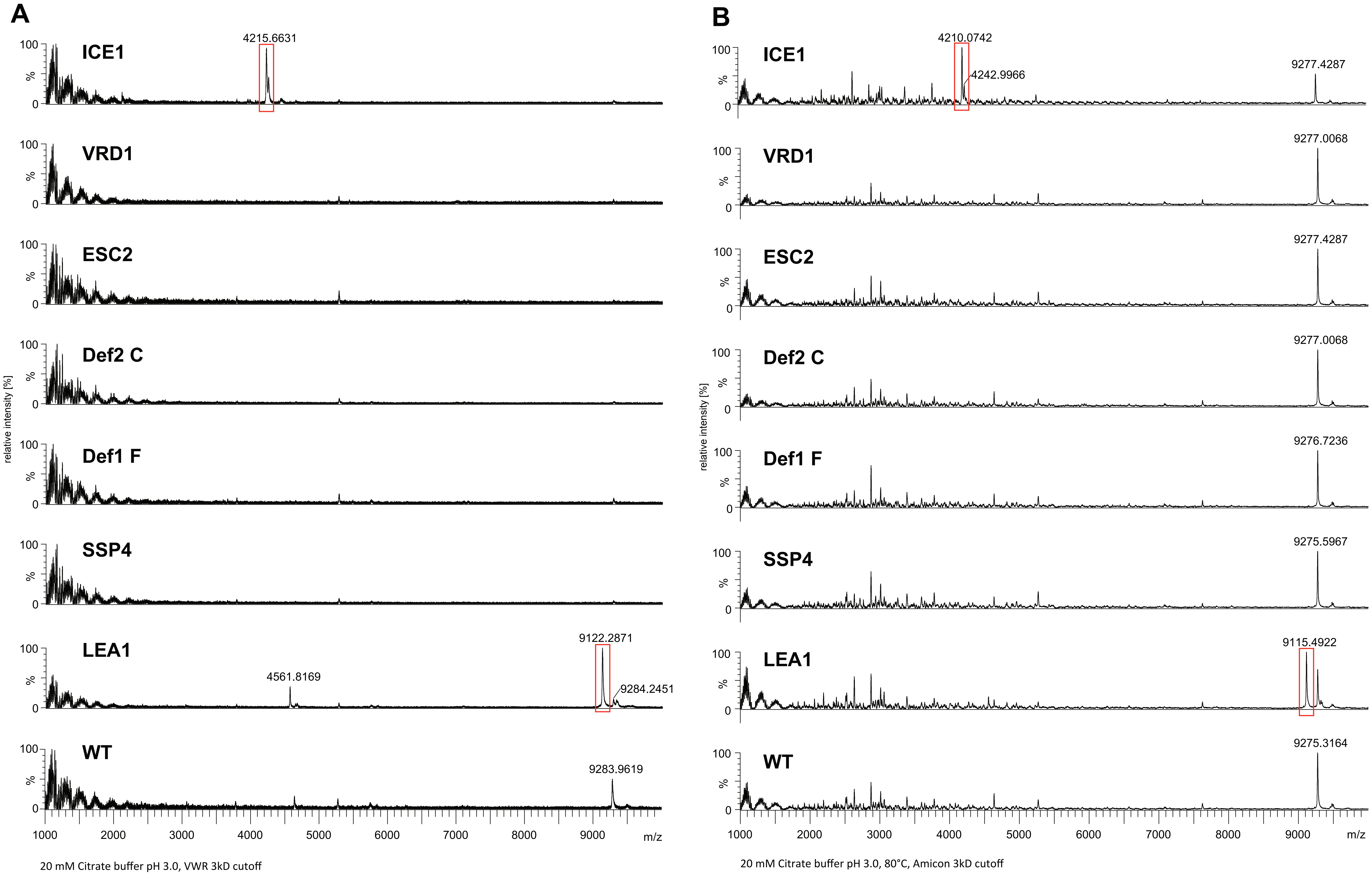

Additional file 2: Comparison of MALDI-TOF mass spectra using different ultrafiltration devices.
Spectra were acquired from the intercellular fluid (ICF) of WT and transgenic plants in the mass range 1–10 kDa. Peaks within the expected mass ranges from the ICE and LEA lines are indicated. **(A)** ICF was extracted with citrate buffer (pH 3.0) and desalted by ultrafiltration (VWR 3K columns). **(B)** ICF was extracted with citrate buffer (pH 3.0), heat treated (80°C) and desalted by ultrafiltration (Amicon 3K columns). MALDI-TOF instrument was operated in linear ion mode.
