# Supplementary material for: Label-free nanoUPLC-MSE based quantification of antimicrobial peptides from the leaf apoplast of Nicotiana attenuata
Source: BMC Plant Biol. 2015 Jan 21;15:18. doi: 10.1186/s12870-014-0398-9 (PMC4318441; doi:10.1186/s12870-014-0398-9)
Supplement: Additional file 3: — Expected masses of AMP peptides after tryptic digest. The peptides (minimum 300 Da) were computed using the Expasy server (http://web.expasy.org/peptide_mass/). Tryptic peptides confirmed by MSE are underlined. [file 12870_2014_398_MOESM3_ESM.docx]

**Additional file 3. Expected masses of AMP peptides after tryptic digest.**

The peptides (minimum 300 Da) were computed using the Expasy server (http://web.expasy.org/peptide_mass/). Tryptic peptides confirmed by MS^E^ are underlined.

| **#line** | **monoisotopic mass** | **position** | **peptide sequence** |
| --- | --- | --- | --- |
| DEF1 | 1885.8571 | 6-22 | AESNTFEGFCVTKPPCR |
| DEF1 | 829.3405 | 42-48 | CICYKPC |
| DEF1 | 567.2773 | 30-34 | FTDGK |
| DEF1 | 438.2017 | 2-5 | STCK |
| DEF1 | 434.2431 | 24-27 | ACLK |
| DEF1 | 401.2871 | 38-40 | ILR |
| DEF1 | 337.1540 | 35-37 | CSK |
| DEF2 | 1863.9091 | 5-21 | TESNTFPGICITKPPCR |
| DEF2 | 894.3774 | 29-36 | FTDGHCSK |
| DEF2 | 767.3249 | 41-47 | CLCTKPC |
| DEF2 | 650.3178 | 23-28 | ACISEK |
| DEF2 | 401.2871 | 37-39 | ILR |
| DEF2 | 379.1646 | 2-4 | ECK |
| VRD | 1294.5588 | 13-24 | CLIDTTCAHSCK |
| VRD | 918.3518 | 39-46 | TCYCLVNC |
| VRD | 811.3767 | 27-34 | GYIGGNCK |
| VRD | 595.2942 | 2-6 | TCMIK |
| VRD | 576.2776 | 8-12 | EGWGK |
| VRD | 464.2286 | 35-38 | GMTR |
| FAB | 1753.7455 | 12-27 | FNGPCLTDTHCSTVCR |
| FAB | 848.3468 | 33-40 | GGDCHGFR |
| FAB | 572.1699 | 43-47 | CMCLC |
| FAB | 553.2616 | 28-32 | GEGYK |
| FAB | 458.3085 | 1-4 | LLGR |
| FAB | 376.1939 | 9-11 | SNR |
| ICE | 1708.6552 | 11-25 | EDQGPPFCCSGFCYR |
| ICE | 716.3838 | 26-31 | QVGWAR |
| ICE | 470.2068 | 32-35 | GYCK |
| ICE | 363.2060 | 2-4 | CIK |
| ICE | 335.1496 | 8-10 | GCR |
| ICE | 318.1772 | 5-7 | NGK |
| PNA | 3022.1537 | 11-40 | LCGNGLCCSQWGYCGSTAAYCGAGCQSQCK |
| PNA | 591.2667 | 1-5 | QQCGR |
| PNA | 518.2681 | 6-10 | QASGR |

(continued)

| **#line** | **average mass** | **position** | **peptide sequence** |
| --- | --- | --- | --- |
| ESC | 886.4992 | 25-32 | EVGLDVVR |
| ESC | 877.4447 | 33-41 | TGIDIAGCK |
| ESC | 857.5454 | 13-20 | NLLISGLK |
| ESC | 551.3187 | 1-5 | GIFSK |
| ESC | 417.2456 | 21-24 | NVGK |
| ESC | 388.2554 | 6-9 | LAGK |
| ESC | 308.0911 | 44-46 | GEC |
| SSP | 886.5145 | 19-26 | FPSIPIGR |
| SSP | 755.3327 | 30-35 | FVQCCR |
| SSP | 682.3341 | 1-6 | SFGLCR |
| SSP | 553.2551 | 10-14 | GFCAR |
| SSP | 365.1602 | 27-29 | CSR |
| SSP | 304.1655 | 37-38 | VW |
| LEA | 1811.8302 | 73-89 | CGVSIPYQISPNTDCSK |
| LEA | 1518.7910 | 57-72 | SYSGINLGNAAGLPGK |
| LEA | 1179.5900 | 11-21 | MAPCLPYVTGK |
| LEA | 963.4928 | 1-10 | AIGCNTVASK |
| LEA | 947.4437 | 22-32 | GPLGGCCGGVK |
| LEA | 878.4223 | 45-52 | QAVCNCLK |
| LEA | 715.4097 | 33-39 | GLIDAAR |
| LEA | 589.2940 | 40-44 | TTPDR |
| LEA | 432.2816 | 53-56 | TLAK |
| CAP | 2361.9602 | 1-28 | GYGGHGGHGGHGGHGGHGGHGHGGGGHG |
| CAP | 3258.3015 | 1-38 | GYHGGHGGHGGGYNGGGGHGGHGGGYNGGGHHGGGGHG |
