# Supplementary material for: Label-free nanoUPLC-MSE based quantification of antimicrobial peptides from the leaf apoplast of Nicotiana attenuata
Source: BMC Plant Biol. 2015 Jan 21;15:18. doi: 10.1186/s12870-014-0398-9 (PMC4318441; doi:10.1186/s12870-014-0398-9)
Supplement: Additional file 4: — Biological and analytical variability of AMPs quantified using nanoUPLC-MS E . The AMP abundance in 3–6 individual biological replicates is shown, each derived from the intercellular fluid extraction of a single N. attenuata plant. Error bars indicate the standard error of 3–5 technical replicates, n.d. = not detected. [file 12870_2014_398_MOESM4_ESM.pdf]

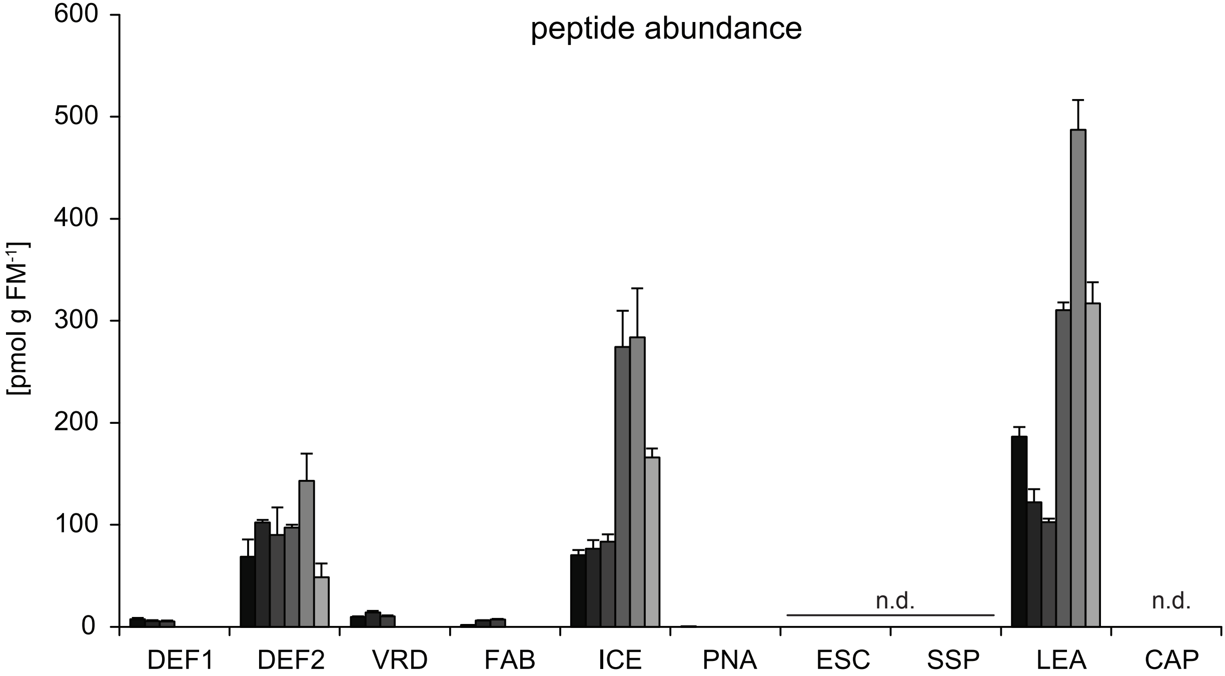

Additional file 4: Biological and analytical variability of AMPs quantified using nanoUPLC-MS^E. The AMP abundance in 3 – 6 individual biological replicates is shown, each derived from the intercellular fluid extraction of a single *N. attenuata* plant. Error bars indicate the standard error of 3 – 5 technical replicates, n.d. = not detected.
