# Supplementary material for: Label-free nanoUPLC-MSE based quantification of antimicrobial peptides from the leaf apoplast of Nicotiana attenuata
Source: BMC Plant Biol. 2015 Jan 21;15:18. doi: 10.1186/s12870-014-0398-9 (PMC4318441; doi:10.1186/s12870-014-0398-9)
Supplement: Additional file 5: — ICF sample composition regarding non-target proteins and impurities. (A) Comparison of the protein composition from total soluble plant extracts vs intercellular fluid (ICF) extracts. Dominant intracellular proteins such as the RuBisCO large subunit (LSU) and small subunit (SSU) are highlighted. ICF extracts (MES buffer pH 5.5) did not indicate the presence of major intracellular contaminations, but were enriched with the fraction of interest (peptides with a mass <10 kDa). (B) SPE peptide desalting removed larger proteins, impurities and enriched basic peptides. (C) Average abundance of the most commonly detected endogenous proteins calculated from LC-MSE data of 11 different genotypes (n = 11, ± SEM) in relation to the AMPs of interest (shown in black). The spiked BSA standard (200 fmol on column) is shown in red. Commonly detected endogenous proteins were apoplastic AMPs (shown in yellow) and chloroplast proteins (shown in green). LTP = Lipid transfer protein. [file 12870_2014_398_MOESM5_ESM.pdf]

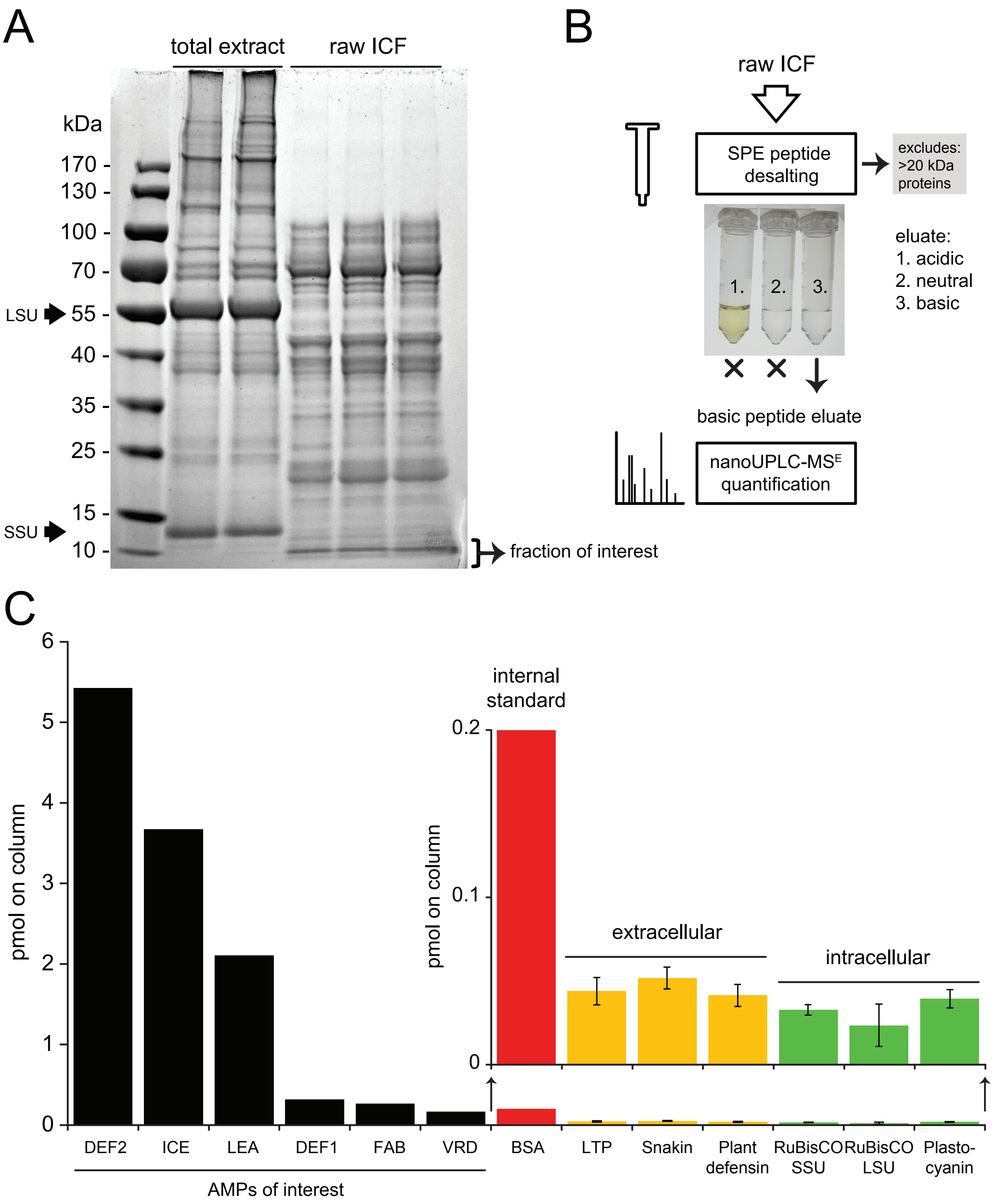

Additional file 5: ICF sample composition regarding non-target proteins and impurities.
(A) Comparison of the protein composition from total soluble plant extracts vs intercellular fluid (ICF) extracts. Dominant intracellular proteins such as the RuBisCO large subunit (LSU) and small subunit (SSU) are highlighted. ICF extracts (MES buffer pH 5.5) did not indicate the presence of major intracellular contaminations, but were enriched with the fraction of interest (peptides with a mass <10kDa). **(B)** SPE peptide desalting removed larger proteins, impurities and enriched basic peptides. **(C)** Average abundance of the most commonly detected endogenous proteins calculated from LC-MS^E data of 11 different genotypes (n = 11, ± SEM) in relation to the AMPs of interest (shown in black). The spiked BSA standard (200 fmol on column) is shown in red. Commonly detected endogenous proteins were apoplastic AMPs (shown in yellow) and chloroplast proteins (shown in green). LTP = Lipid transfer protein
