# Supplementary material for: Label-free nanoUPLC-MSE based quantification of antimicrobial peptides from the leaf apoplast of Nicotiana attenuata
Source: BMC Plant Biol. 2015 Jan 21;15:18. doi: 10.1186/s12870-014-0398-9 (PMC4318441; doi:10.1186/s12870-014-0398-9)
Supplement: Additional file 6: — Determination of AMP abundance in the supernatant. (A) The supernatants after vacuum infiltration (MES, pH 5.5) were SPE desalted, spiked with BSA and analyzed using nanoUPLC-MSE, n.d. = not detected; (B) Comparison of all peptides from the supernatant of the respective genotype. (C) Comparison of DEF1 abundance in the supernatant. (D) Comparison of DEF2 abundance in the supernatant. [file 12870_2014_398_MOESM6_ESM.pdf]

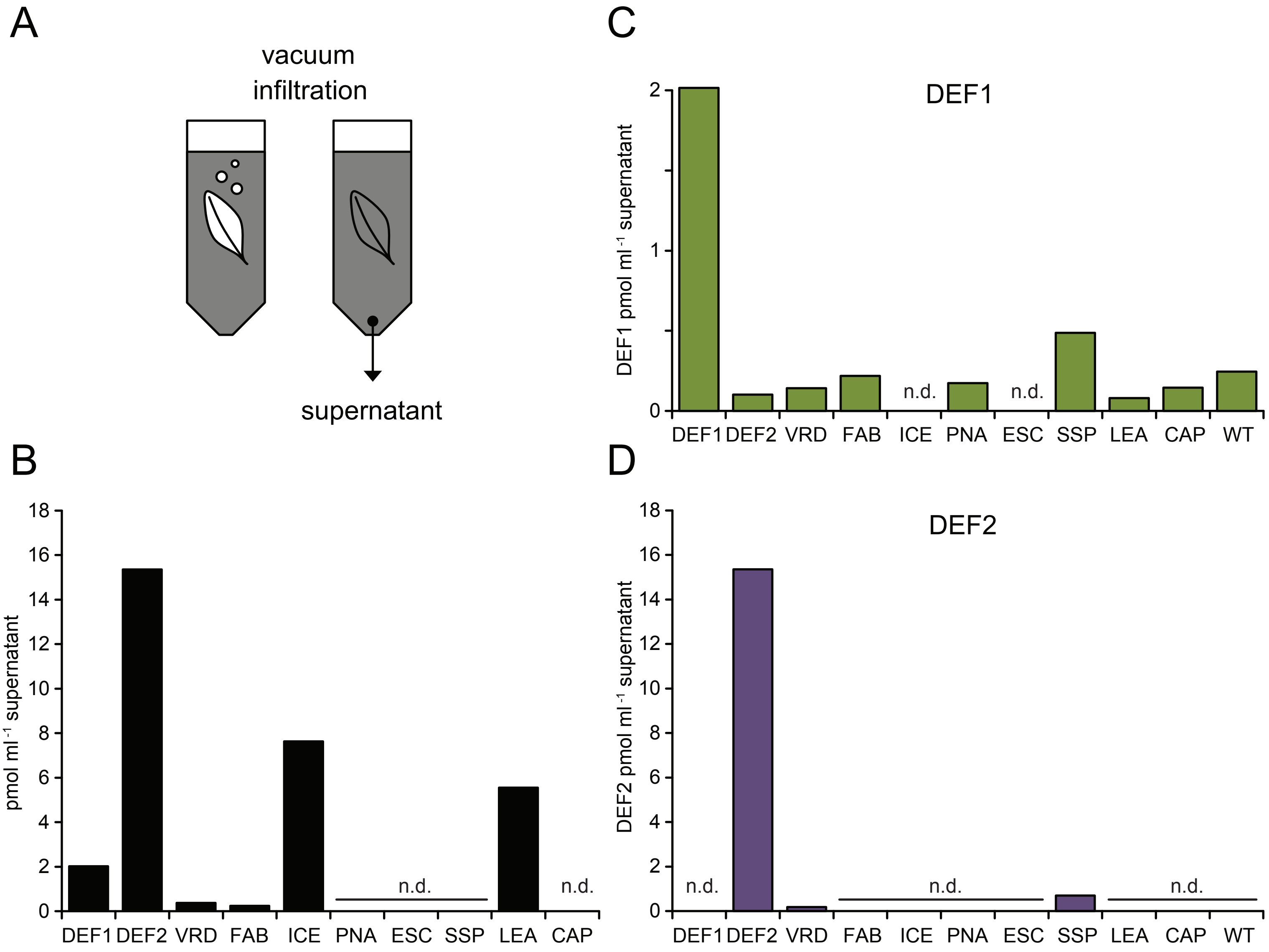

Additional file 6: Determination of AMP abundance in the supernatant.
(A) The supernatants after vacuum infiltration (MES, pH 5.5) were SPE desalted, spiked with BSA and analyzed using nanoUPLC-MS^E, n.d. = not detected; **(B)** Comparison of all peptides from the supernatant of the respective genotype. **(C)** Comparison of DEF1 abundance in the supernatant. **(D)** Comparison of DEF2 abundance in the supernatant.
